# Subdiagnosis, but not presence of vestibular symptoms, predicts balance impairment in migraine patients – a cross sectional study

**DOI:** 10.1186/s10194-020-01128-z

**Published:** 2020-05-24

**Authors:** Letícia Zorzin, Gabriela F. Carvalho, Jens Kreitewolf, Roberto Teggi, Carina F. Pinheiro, Jéssica R. Moreira, Fabíola Dach, Débora Bevilaqua-Grossi

**Affiliations:** 1 Department of Health Sciences, Ribeirão Preto Medical School, University of São Paulo, Av. Bandeirantes, 3900 – Vila Monte Alegre, Ribeirão Preto, SP 14049-900 Brazil; 2 Department of Psychology, University of Lübeck, Lübeck, Germany; 3 Department of Ear, Nose and Throat, San Raffaele University Hospital, Milan, Italy; 4 Department of Neurosciences and Behavioral Sciences, Ribeirão Preto Medical School, University of São Paulo, Ribeirão Preto, Brazil

**Keywords:** Migraine, Dizziness, Vestibular migraine, Postural balance, Headache

## Abstract

**Background:**

Vestibular symptoms and balance changes are common in patients with migraine, especially in the ones with aura and chronic migraine. However, it is not known if the balance changes are determined by the presence of vestibular symptoms or migraine subdiagnosis. Therefore, the aim of this study was to verify if the migraine subdiagnosis and/or the presence of vestibular symptoms can predict balance dysfunction in migraineurs.

**Methods:**

The study included 49 women diagnosed with migraine with aura, 53 without aura, 51 with chronic migraine, and 54 headache-free women. All participants answered a structured questionnaire regarding migraine features and presence of vestibular symptoms, such as dizziness/vertigo. The participants performed the Modified Sensory Organization Test on an AMTI© force plate. The data were analysed using a linear mixed-effect regression model.

**Results:**

The presence of vestibular symptoms did not predict postural sway, but the subdiagnosis was a significant predictor of postural sway. Migraine with aura patients exhibited more sway than migraine patients without aura when the surface was unstable. Additionally, we found high effect sizes (ES > 0.79) for postural sway differences between patients with chronic migraine or with aura compared to controls or migraine without aura, suggesting that these results are clinically relevant.

**Conclusions:**

The subdiagnosis of migraine, instead of the presence of vestibular symptoms, can predict postural control impairments observed in migraineurs. This lends support to the notion that balance instability is related to the presence of aura and migraine chronicity, and that it should be considered even in patients without vestibular symptoms.

## Introduction

Migraineurs present a high prevalence of vestibular symptoms, such as dizziness and vertigo [1–10]. The co-occurrence of these symptoms with migraine is more frequent than expected by chance [10, 11] during both the ictal and interictal period [6, 9], and it can augment the impact on migraineurs’ quality of life [1, 4]. In that way, these symptoms can be considered as either part of the migraine presentation [11] or a different classification – as described in the Appendix of the International Classification of Headache Disorders (ICHD-III) as vestibular migraine [12], which was jointly formulated with the Committee for Classification of Vestibular Disorders of the Bárány Society [13]. Patients with chronic migraine and migraine with aura present more prevalence of vestibular symptoms compared to migraineurs without aura [1–6, 9]. Furthermore, it has been suggested that the presence of aura and headache frequency can predict dizziness handicap [4].

In addition to vestibular symptoms, migraineurs often present balance disorders [5, 7, 9, 14–18], which is also augmented by the presence of aura and chronicity in the interictal [5–7] and ictal period [19]. Without considering the migraine subdiagnosis, some studies suggest that migraineurs with vestibular symptoms present more balance instability [15, 20] in association with [20] or without otoneurologic alterations [15]. On the contrary, it is also evidenced no balance neither otoneurologic differences between patients with and without vestibular symptoms [6, 21–25].

The etiology of the disequilibrium in migraineurs is not yet established, but it can be related to the presence of subclinical vestibular and cerebellar dysfunctions, which is verified by otoneurologic alterations and subclinical ischemic-like lesions in the vestibular and cerebellar regions [23, 24, 26–29]. These structural brain alterations are also more prevalent in patients with migraine with aura or chronic migraine [26–28].

Taking all together, it is not clear if the statements of worse balance in patients with vestibular migraine or migraine-associated dizziness are influenced by the migraine subdiagnosis, since patients with aura and chronic migraine present a high prevalence of structural brain alterations [26–28], balance impairments [5–7], vestibular symptoms [1–5] and dysfunctions [20]. To improve our understanding regarding the migraine spectrum and to ensure adequate classification and subsequent treatment, it is important to determine whether the balance instability of these patients is determined by the presence of vestibular symptoms or by the migraine features itself [15, 20]. Therefore, our aim was to investigate the factors that could explain the balance alterations in migraine patients, including dizziness or vertigo, presence of aura and chronicity of the migraine. We hypothesize that the migraine subdiagnosis, and not the presence of vestibular symptoms, can predict the balance impairment in these patients.

## Material and methods

We screened 153 migraineurs from a tertiary outpatient headache clinic at the Clinics Hospital of Ribeirão Preto, Brazil, who accepted to participate in the study. These patients were diagnosed with migraine by expert neurologists in headaches and were classified into migraine without aura (*n* = 53), with aura (*n* = 49) or chronic migraine (*n* = 51) according to the ICHD-III criteria [12]. Furthermore, 54 headache-free individuals were included as controls.

Women aged between 18 and 55 years and with at least 3 episodes of migraine in the last 3 months were included in this study. Patients were excluded if they presented any previously diagnosed vestibular or systemic diseases (i.e., fibromyalgia, non-controlled arterial hypertension, rheumatoid arthritis or diabetes mellitus), abnormal neurological exam, body mass index (BMI) over 30, any musculoskeletal disorder that could influence the tests, concomitant headaches, or presence of headache during evaluation. Furthermore, patients in use of any medication prescribed for vertigo or dizziness treatment such as meclizine, flunarizine, cinnarizine, betahistine, and/or benzodiazepinics were excluded. The same inclusion and exclusion criteria, further than the absence of any sort of headache, was applied for the control group.

All migraine patients were under standard and tailored care at the outpatient headache clinic. According to the service protocol, the best abortive or prophylactic medication were prescribed. The prescribed drug classes could include beta-blockers, antidepressants, serotonin norepinephrine reuptake inhibitors or antiepileptic drugs.

A structured questionnaire was administered to obtain the demographic data and the migraine features, including age, body mass index (BMI), migraine onset and frequency, duration of the migraine attack, pain intensity (numeric pain rating scale NPRS, 0–10) and presence of vertigo and dizziness.

Dizziness and vertigo were defined according to the International Classification of Vestibular Disorders of the Bárány Society. In which dizziness is defined as the sensation of disturbed or impaired spatial orientation without a false or distorted sense of motion; and vertigo is the sensation of self-motion (of head/body) when no self-motion is occurring, or the sensation of distorted self-motion during a normal head movement, or the false sensation that the visual surround is spinning or flowing [30].

The posturography has been widely used to assess postural control also in migraineurs [5, 6, 9, 16–18] and allow the reliable and valid measurement of the sensory information integration [21, 31, 32]. The subjects were instructed to perform the Modified Sensory Organization Test (modified SOT) and their balance was measured by means of the displacement area (cm^2^) from an AMTI© force plate (OR6-7-1000, 100 Hz, Watertown MA, USA). The Modified SOT is composed by four conditions of stable and unstable surfaces, with open and closed eyes. The unstable support was performed using a 20x50x50cm foam over the force place with 0,5 kg/cm^3^ of density. Patients were instructed to stay in upright position over the force plate with arms relaxed along the body, feet 15 cm apart, gazing a target in the high of their eyes positioned 2 m away [5, 31]. Each condition was repeated 3 times for 30 s [5, 31].

The study was approved by the University of São Paulo local ethics committee (protocol number 16693/2012), and all patients signed the informed consent form before the evaluation.

### Statistical analysis

The sample size was defined based on a previous similar study [6], which considered the same assessment and same migraine subdiagnosis with 35 participants for each group. In order to include another variable of interest in the same analysis (presence of dizziness/vertigo), we increased up to 50% of the sample size from a convenience sampling.

All statistical analyses were performed in R (R Core Team, 2018) using R Studio (version 1.1.447) [33], with the aim of investigating whether the subdiagnosis and/or the presence of vestibular symptoms, is a significant predictor for postural sway in patients with migraine. We, therefore, tested the effects of subdiagnosis and vestibular symptoms across different balance assessment conditions (firm and foam surface, open and closed eyes) by fitting linear mixed-effect models to sway measured on single trials.

Similar to several of our previous studies [34–36], we followed an iterative model-fitting procedure: Starting with the minimal model that only included the subject-specific random intercepts, we first added fixed- and then random-effects terms in a step-wise fashion. Fixed-effect terms were added in the order of their clinical importance (i.e., diagnosis, vestibular symptoms, surface, eyes, and interactions between these factors). Note that this did not include the diagnosis-by-vestibular symptoms interaction because the model was not full factorial with regard to these two factors (i.e., none of the control subjects had vestibular symptoms).

To model the potential effects of vestibular symptoms, surface, and eyes (open vs closed) on sway, we used deviation coding. This means that each level of these factors was compared to the grand average. For diagnosis, we used a simple coding scheme in which the control group was defined as reference level. The random-effect terms included subject-specific random slopes for the main factors surface and eyes as well as the interaction between surface and eyes. After each step, we fitted the model using maximum-likelihood estimation, and tested whether the inclusion of single model terms significantly improved the model fit using likelihood-ratio tests. Terms that significantly improved the model fit were kept in the model, nonsignificant terms were dropped, resulting in a best-fitting model.

To obtain *p*-values for individual terms of the best-fitting model, we used the Satterthwaite approximation for degrees of freedom [37], and a significance level of 5% was set. Due to the incremental model fitting produce, some fixed-effects terms did not yield significance in the best-fitting model. However, their inclusion in the best-fitting model was warranted by the significant improvement in model fit as assessed by likelihood-ratio tests during the model fitting procedure (see above). We additionally calculated Bayes Factors (BFs) for the main factors of interest, diagnosis and dizziness, using the Bayes Factor package [38]. The BF indicates how many times more likely the observed data are under the more complex model (here, the best-fitting model) than under the simpler model (here, best-fitting model excluding a particular model term of interest). A BF of 0.33 or smaller is interpreted as providing evidence in favor of the null hypothesis (the factor of interest does not improve the model fit), whereas a BF of 3 or larger is interpreted as evidence against it [39].

To explore significant interaction terms, we performed post-hoc comparisons using Tukey’s range tests, as implemented in the lsmeans package [40]. We report unstandardized coefficients *b* to provide an estimate of effect size for fixed effects. Additionally, we convert these coefficients to Cohen’s *d* to enhance interpretability and to allow for comparison with previous publications [9, 41]. Cohen’s *d* values around 0.20 were interpreted as small, 0.50 as medium, and 0.80 as large effects [42].

## Results

Up to 207 subjects fulfilled the inclusion and exclusion criteria and therefore were included in the study. The demographic data and the migraine features of groups are described in Table 1.

**Table 1:** Demographic data of the sample. Average data are reported (with standard deviation in parentheses) unless otherwise indicated

	CG (*n* = 54)	MoA (*n* = 53)	MA (*n* = 49)	CM (*n* = 51)	*p*
Age (years)	34.03 (9.84)	32.22 (9.63)	35.73 (9.3)	35.72 (9.48)	0.18
BMI (km/m^2^)	24.4 (3.9)	24.21 (4.84)	26.0 (4.5)	25.13 (4.15)	0.12
Migraine onset (years)	–	14.64 (9.59)	18.67 (11.13)	17.64 (9.66)	0.09
Migraine frequency (monthly)	–	5.86 (8.91)	6.12 (3.40)	20.66 (8.71)	**< 0.001**^**a**^
Attack duration (hours)	–	14.01 (29.78)	31.45 (31.72)	24.70 (34.53)	**0.01**^**b**^
Pain intensity (NRS: 0–10)	–	7.50 (1.66)	8.2 (1.4)	7.96 (1.58)	0.06
Dizziness/vertigo (%)	0	66	93	84	**< 0.001**^**c**^

*CG* control group, *MoA* migraine without aura group, *MA* migraine with aura group, *CM* chronic migraine group, *BMI* body mass index, *NRS* numeric rating scale. Significant *p*-values are marked by boldface. ^a^CM versus MA and MoA, ^b^MA versus MoA, ^c^Chi-Square test

The iterative model-fitting procedure (see ‘Statistical Analyses’ for details) yielded the following best-fitting model:

$$ lmer\ \Big(\mathrm{Sway}\sim 1+\mathrm{Diagnosis}\ast \mathrm{Surface}\ast \mathrm{Eyes}+\mathrm{Dizziness}+\mathrm{Dizziness}:\mathrm{Eyes}+\left(1+\mathrm{Surface}\ast \mathrm{Eyes}|\mathrm{subject}\right) $$

The results of F-tests for individual fixed-effects terms are summarized in Table 2. These results show that vestibular symptoms were not a significant predictor of sway (*F*_1,206.37_ = 2.51; *p* = 0.114; *BF* = 0.082). On the other hand, migraine diagnosis was a significant predictor (*F*_3,218.43_ = 3.14; *p* = 0.026; *BF* = 42.45). Additionally, the BFs provide strong evidence for the *presence* of an effect of diagnosis on sway as well as strong evidence for the *absence* of an effect of dizziness on sway. The significant interaction between diagnosis and surface (*F*_3,206.86_ = 4.22; *p* = 0.006) shows that the effect of diagnosis on sway depended on surface condition.

**Table 2:** Results of F-tests for individual fixed-effects terms using Satterthwaite approximation for degrees of freedom

Fixed effect	Sum Squares	df (num., den.)	F value	*P* value
Diagnosis	174.5	3, 218.35	3.1426	**0.026**
Surface	10,813.4	1, 206.91	584.3510	**< 0.001**
Eyes	4402.0	1, 225.33	237.8827	**< 0.001**
Vestibular Symptoms	46.4	1, 206.25	2.5078	0.11
Diagnosis:Surface	234.6	3, 206.91	4.2255	**0.006**
Diagnosis:Eyes	74.8	3, 234.80	1.3475	0.25
Eyes:Vestibular Symptoms	37.3	1, 262.60	2.0182	0.15
Diagnosis:Surface:Eyes	90.0	230.05	1.6218	0.18

Significant *p*-values are marked by boldface. The best-fitting model, including random-effects terms, had an R^2^ of 0.84

**Fig. 1:**
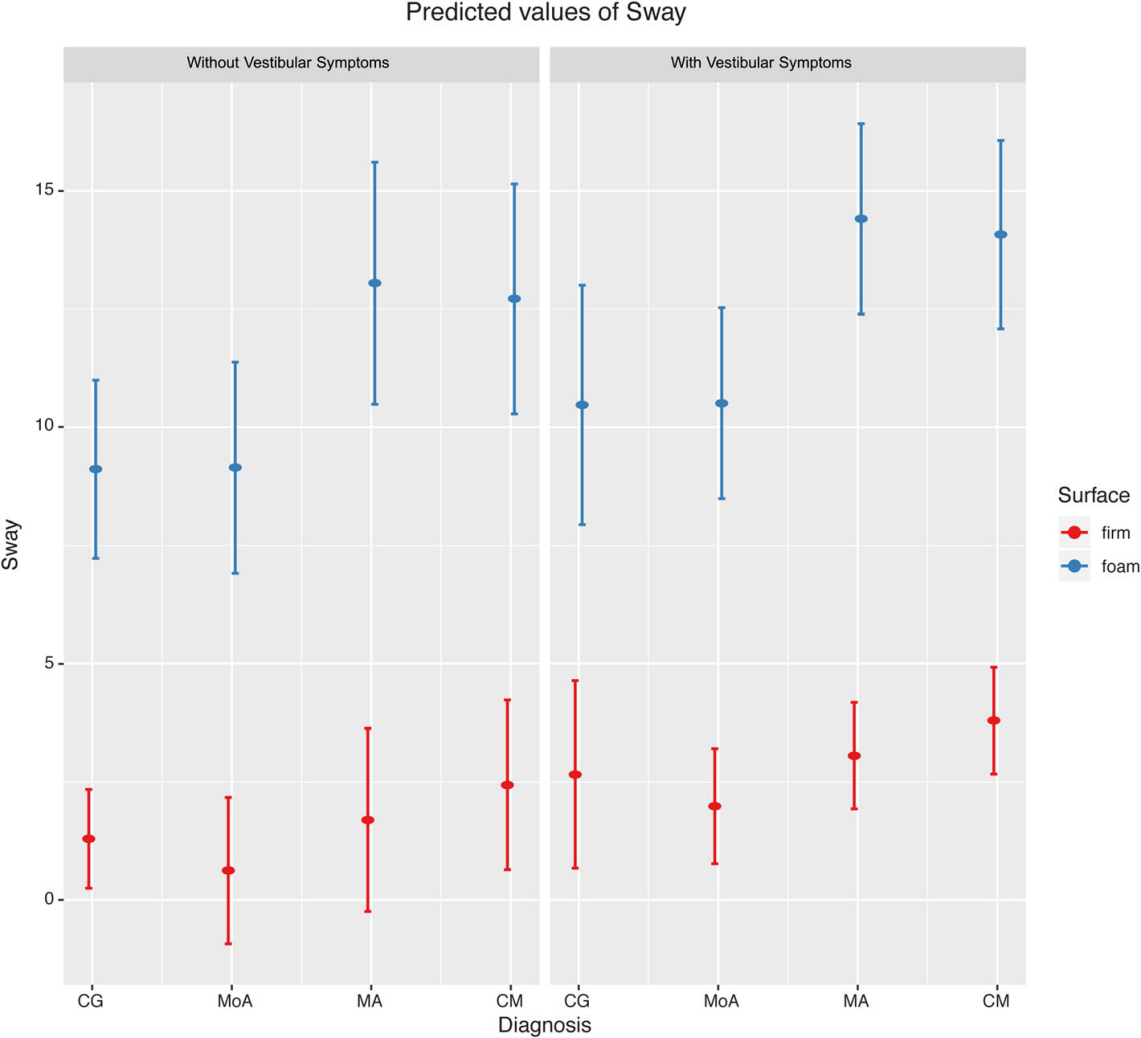
Predicted sway based on the model with and without vestibular symptoms. Left: vestibular symptoms absent; Right: vestibular symptoms present

To explore the diagnosis-by-surface interaction, we computed pairwise comparisons between diagnosis groups in each surface condition. The results of these post-hoc tests are summarized in Table 3. The only significant difference was observed between the MoA and MA groups in the foam surface condition (*t*_212.33_ = − 2.70; *p* = 0.037; *b* = − 3.90), showing that, migraine patients with aura exhibit more sway on foam surface than migraine without aura. Similarly large, albeit non-significant, effects were observed under the foam surface condition between controls and migraine patients with aura (*t*_275.58_ = − 2.42; *p* = 0.075; *b* = − 3.94), controls and patients with chronic migraine (*t*_266.39_ = − 2.29; *p* = 0.103; *b* = − 3.60), as well as migraine patients without aura and patients with chronic migraine (*t*_207.97_ = − 2.51; *p* = 0.061; *b* = − 3.56). Estimated group differences were considerably smaller under the firm surface condition (unstandardized coefficients, *b*s, ranged between − 1.81 and 0.67) (Table 3, Fig. 1).

**Table 3:** Results of post-hoc Tukey’s range tests for the diagnosis-by-surface interaction

	Firm surface						Foam Surface					
	Estimate *b*	SE	df	t	*P* value	Cohen’s *d*	Estimate *b*	SE	df	t	*P* value	Cohen’s *d*
CG versus MoA	0.6709	0.956	205	0.702	0.89	−1.08	−0.0339	1.492	247	−0.023	1.00	0.04
CG versus MA	−0.3977	1.129	205	−0.352	0.98	**0.57**	−3.9350	1.625	276	−2.421	0.075	**3.67**
CG versus CM	−1.1396	1.064	205	−1.071	0.70	**1.72**	−3.6029	1.572	266	−2.292	0.10	**3.43**
MoA versus MA	−1.0686	0.828	204	−1.291	0.70	**1.71**	−3.9012	1.445	212	−2.700	**0.037**	**3.73**
MoA versus CM	−1.8105	0.799	204	−2.265	0.10	**3.03**	−3.5691	1.419	208	−2.515	0.060	**3.49**
MA versus CM	−0.7419	0.803	204	−0.924	0.79	**1.14**	0.3321	1.440	205	0.231	0.99	−0.31

Significant differences across diagnosis groups and *significant* effect sizes [41] are marked by boldface. *CG* control group, *MoA* migraine without aura, *MA* migraine with aura, *CM* chronic migraine

Taken together, these results provide evidence that diagnosis, but not the presence of vestibular symptoms, predicts postural sway in migraine patients. Importantly, meaningful differences across subdiagnosis of migraine groups were only observed under foam, but not firm, surface condition.

## Discussion

Our aim was to investigate the factors that could predict balance impairments in migraine patients, including dizziness or vertigo, presence of aura and chronicity of the migraine. We hypothesized that the balance changes would be determined by the migraine subdiagnosis, and not by the presence of vestibular symptoms. Our results demonstrated that the presence of vestibular symptoms does not predict the postural sway of migraineurs. On the other hand, the subdiagnosis, especially of migraine with aura, can predict the imbalance of these patients. These results are more evident in challenging situations, such as on the unstable surface. Furthermore, these results were confirmed by the Bayes Factors analysis, which demonstrated evidence in favor of the diagnosis hypothesis, and evidence against the vestibular symptoms hypothesis.

There was a significant interaction between diagnosis and the surface condition in our results; that is, migraine patients with and without aura differed in sway on unstable surface only. Although we only found a significant difference between these two migraine groups in the unstable surface condition, the effect-sizes for other pairwise comparisons were similar in magnitude; e.g., between controls or migraine without aura versus chronic migraine and migraine with aura (Fig. 1, Table 3). These results agree with previous published studies [5–7].

The presence of migraine is related to vestibular and cerebellar dysfunctions, verified by subclinical ischemic-like lesions in these regions [23, 24, 26–29]. It is indeed speculated that the cortical spreading depression – known as the migraine aura generator – can exacerbate the neuronal damage during the migraine attacks [43–45]. Further than correlated with the presence of aura [28], the extension of the ischemic-like lesions increases with the frequency of pain episodes [27]. These statements are in line with our findings, where greater impairment was verified in patients with high migraine frequency (chronic migraine) and aura.

The relationship between vestibular migraine and postural control is controversial. Some studies did not find any alteration in postural balance when comparing groups with migraine with or without dizziness or vertigo [6, 21, 22, 24, 25]. Other studies, however, found a relation between the presence of vestibular symptoms and worse postural balance [15, 20]. Regardless of these previous data, none of these studies investigated the impact of the vestibular symptoms on postural control considering the influence of aura and migraine chronicity. The present study is the first to analyze the presence of vestibular symptoms and the subdiagnosis contribution in the same statistical model to verify the influence of both in postural control.

Another point is that vestibular symptoms are unspecific and could be present with or without vestibular dysfunction [15, 20, 24], which would influence the results of postural balance. In the same way, some migraineurs without self-reported vestibular symptoms also present vestibular dysfunction [23], suggesting that these vestibular problems and symptoms can be related to the presence of migraine and probably not related to a different condition. Furthermore, the vestibular symptoms are more prevalent in migraine with aura and chronic migraine [1–5], even in the interictal period [4, 6, 9]. This implies that an overlap between these subdiagnosis and the presence of the vestibular symptoms may occur. This overlapping may be considered a confounding factor, and may require further investigation.

Our study suggests that migraineurs with vestibular symptoms exhibit similar postural control than migraineurs without self-report of vestibular symptoms. Additionally, we verified a high prevalence of the vestibular symptoms among the patients, which agrees with previous findings [1–11]. Based on the present and previous findings, we suggest that the vestibular symptoms and balance changes are a common condition among migraineurs and may be present on different severity levels according to the classic clinical presentation of migraine; and not due to a different classification considering only the presence of vestibular symptoms.

The vestibular migraine diagnosis is essentially based on the self-report of the vestibular symptoms [12]. However, the presence of these symptoms can be influenced by several factors, including emotional aspects such as depression or anxiety [46]; and according to the migraine presentation, such as aura symptoms or higher frequency of migraine attacks [1–5]. Furthermore, there is controversial evidence that neuro-vestibular examination including resonance imaging or otoneurologic tests would provide evidence of vestibular migraine diagnosis [20, 24, 47–49]. Ongun et al. [21] suggest that the posturography can be useful to demonstrate involvement of the vestibular and somato-sensorial systems in these patients, since it can assess the somatosensory, vestibular, and visual information objectively.

Along with previous studies, our results provide further evidence for the absence of a difference in postural control among patients with and without vestibular symptoms. In addition, the standard acute and prophylactic treatment for migraine can improve the vestibular symptoms [50]. So, perhaps it might not be necessary to classify these patients as another migraine subtype, since there are no substantial differences regarding examination or treatment.

One of the limitations of this study is that although our patients presented self-reported vestibular symptoms, the vestibular migraine criteria was not applied. Therefore, comparisons between patients diagnosed with and without vestibular migraine diagnosis are not possible. It is well known that the presence of anxiety or depression is related to higher prevalence of vestibular disorders and could be a trigger to these disorders, including vestibular migraine [46], and therefore psychological disorders such as anxiety or depression disorders should be considered for futures studies. Additionally, the results can be overestimated since our sample was recruited from a tertiary hospital and can be generalized just to female migraineurs.

Despite these limitations, this is the first study that verified the ability of balance prediction in migraineurs according to the presence of vestibular symptoms and migraine subdiagnosis. Furthermore, the patients were diagnosed by neurologists specialized in headache, and a large sample was collected, reflecting the external validity and clinical relevance of this study.

## Conclusion

The present study verified that migraine patients with and without vestibular symptoms do not differ from one another since no balance changes were verified; instead, migraine subdiagnosis, especially migraine with aura, can be considered a predictor factor for balance changes. Furthermore, our results suggest that the balance evaluation and treatment among patients with migraine provides an important clinical tool even in those patients without self-report of vestibular symptoms. Finally, the present study may trigger a comprehensive evaluation regarding the balance aspects in this sample for future studies and therapy possibilities. Also, further studies of postural control and vestibular assessment should be performed in patients diagnosed with and without vestibular migraine.

## Data Availability

All data generated or analysed during this study are included in this published article.
